# Heat Acclimation in Females Does Not Limit Aerobic Exercise Training Outcomes

**DOI:** 10.3390/ijerph19095554

**Published:** 2022-05-03

**Authors:** Mark L. McGlynn, Christopher Collins, Walter Hailes, Brent Ruby, Dustin Slivka

**Affiliations:** 1 School of Health and Kinesiology, University of Nebraska at Omaha, Omaha, NE 68182, USA; markmcglynn@unomaha.edu (M.L.M.); christophercollins@unomaha.edu (C.C.); 2 School of Integrative Physiology and Athletic Training, University of Montana, Missoula, MT 59812, USA; walter.hailes@mso.umt.edu (W.H.); brent.ruby@mso.umt.edu (B.R.)

**Keywords:** thermoregulation, mRNA, gene expression, mitochondrial biogenesis, VO_2peak_, *PGC-1α*

## Abstract

Recent aerobic exercise training in the heat has reported blunted aerobic power improvements and reduced mitochondrial-related gene expression in men. It is unclear if this heat-induced blunting of the training response exists in females. The purpose of the present study was to determine the impact of 60 min of cycling in the heat over three weeks on thermoregulation, gene expression, and aerobic capacity in females. Untrained females (n = 22; 24 ± 4yoa) were assigned to three weeks of aerobic training in either 20 °C (n = 12) or 33 °C (n = 10; 40%RH). Maximal aerobic capacity (39.5 ± 6.5 to 41.5 ± 6.2 mL·kg^−1^·min^−1^, *p* = 0.021, η_p_^2^ = 0.240, 95% CI [0.315, 3.388]) and peak aerobic power (191.0 ± 33.0 to 206.7 ± 27.2 W, *p* < 0.001, η_p_^2^ = 0.531, 95% CI [8.734, 22.383]) increased, while the absolute-intensity trial (50%VO_2peak_) HR decreased (152 ± 15 to 140 ± 13 b·min^−1^, *p* < 0.001, η_p_^2^ = 0.691, 95% CI [15.925, 8.353]), but they were not different between temperatures (*p* = 0.440, *p* = 0.955, *p* = 0.341, respectively). Independent of temperature, Day 22 tolerance trial skin temperatures decreased from Day 1 (*p* = 0.006, η_p_^2^ = 0.319, 95% CI [1.408, 0.266), but training did not influence core temperature (*p* = 0.598). Average sweat rates were higher in the 33 °C group vs. the 20 °C group *(p* = 0.008, η_p_^2^ = 0.303, 95% CI [67.9, 394.9]) but did not change due to training (*p* = 0.571). Pre-training *PGC-1α* mRNA increased 4h-post-exercise (5.29 ± 0.70 fold change, *p* < 0.001), was lower post-training (2.69 ± 0.22 fold change, *p* = 0.004), and was not different between temperatures (*p* = 0.455). While training induced some diminished transcriptional stimulus, generally the training temperature had little effect on genes related to mitochondrial biogenesis, mitophagy, and metabolic enzymes. These female participants increased aerobic fitness and maintained an exercise-induced *PGC-1α* mRNA response in the heat equal to that of room temperature conditions, contrasting with the blunted responses previously observed in men.

## 1. Introduction

Current exercise recommendations apply specifics to timing, frequency, intensity, and type of exercise in order to produce optimal chronic adaptations [1]. However, other exercise attributes i.e., ambient temperature, which can have an influence over acute and chronic exercise adaptations, are often overlooked. Hence, there is a need to better define the influence of heat acclimation, defined as the short-term (≤5 days) and/or long-term (>10 days to many weeks) physiological alterations to heated conditions with the goal of improving performance [2]. However, best practices to optimize heat acclimation are still unclear. Although previous evidence suggests that exercising while enduring heat stress has a detrimental effect [3,4], the majority show favorable alterations [4,5,6,7,8,9,10,11]. Acute extended exercise in the heat can impede performance by altering the extracellular fluid (reduced plasma volume) and amplifying cardiovascular stress (increased heart rate). These detrimental influences result in a reduced ability to compensate for the ambient conditions and a steeper rise in core temperature, leading to impaired performance and a sub-optimal training day [3,11,12]. Heat acclimation can directly combat these detrimental physiological alterations [3,5,6,7,8,9,10,11]. After acclimation, evidence suggests that an increase in plasma volume [3,5,7,8,9] allows for an attenuated strain on the cardiovascular system and a reduced submaximal heart rate [3,6,8,11]. Others have observed improved heat dissipation via improved sudomotor sensitivity and sweat output [13] and a diminished rise in core temperature in the heat. More importantly, most of these heat acclimation conclusions are based on data collected from trained and untrained males. Few have investigated how these recommendations may change when applied to female physiology [2,7,14,15]. Recent reviews support substantial physiological differences in thermoregulation between the sexes [16,17,18]. Specifically, greater insulation via an increased body fat percentage, reduced body surface area, and reduced sudomotor function [7,15,19,20] allow for greater heat loss requirements in women (i.e., an elevated set point) compared to men. Therefore, females may require an extended heat acclimation exposure to achieve the same classical heat acclimation responses found in males [21], or females may just acclimate in a non-classical fashion. Indeed, further research is needed to explore the influences of heat acclimation within females.

An acute bout of aerobic exercise can initiate strong mitochondrial-related gene expression associated with positive mitochondrial development [11,22,23,24]. Increased gene expression of *PGC-1α* is the initial step toward multiple downstream alterations to enhance mitochondrial biogenesis and thus aerobic power [25]. By linking together multiple acute exercise sessions, one can demonstrate the potential for chronic molecular adaptations to the applied stressors. Further, by examining gene expression related to mitochondrial biogenesis (*PGC-1α*, *NRF1*, *GABPA*, *VEGF*, *TFAM*, and *ESRRa*), mitophagy (*PINK2*, *PARK2*, *BNIP3*, and *BNIP3-L*), and genes related to the production of metabolic enzymes (*CS*, *COX4*, and *BHAD*), one can gain insight into the initial adaptive improvements due to acute exercise, over training, and heat acclimation. However, an altered gene expression does not necessarily translate to an equivalent protein translation, hence there is a need for functional performance testing (i.e., VO_2peak_). Recent heat acclimation-associated diminished gene expression responses were validated by attenuated aerobic performance outcomes when compared to similar men exercising in temperate conditions [11]. However, it is unclear how these diminished responses, due to training in the heat, will translate to female physiology.

Based on known sex related thermoregulatory differences [15,16,18], it is unknown whether females will also demonstrate a dampened aerobic capacity and gene expression adaptive potential. Therefore, the purpose of this investigation is to determine the impact of 3 weeks of aerobic exercise in the heat (33 °C), compared to room temperature (20 °C), on thermoregulation, aerobic capacity, and gene expression related to mitochondrial biogenesis, mitophagy, and metabolic development in women.

## 2. Materials and Methods

### 2.1. Experimental Design

Twenty-four untrained females (age, 24 ± 4 yrs; height, 167.2 ± 7.6 cm) who did not engage in regular aerobic exercise and volunteered by completing the Institutional Review Board approved informed consent document guided by the ethical principles outlined in the Declaration of Helsinki. Subjects were considered healthy and free of any disease that may interfere with exercise training and/or thermoregulation (i.e., Raynaud’s Syndrome) as assessed via an approved questionnaire. Selection bias was minimized via the application of a concealed random sequence (generated via MS Office Excel) to sort participants into one of two counterbalanced temperature conditions (20 °C or 33 °C, 40% relative humidity). All available attempts to reduce the likelihood of bias were applied; however, the assigned temperature conditions prevented potential performance bias. The design consisted of two (pre- and post-training) fitness assessment visits conducted 3 days before (Day −3) and 3 days after (Day 25) the completion of the 3-week training protocol. To examine the subject’s acclimation status, a fixed-intensity cycling temperature tolerance trial, conducted at 50%Wpeak, was conducted within the assigned temperature condition (20 °C or 33 °C) on Day 1 (pre) and Day 22 (post). Therefore, Day 1 was dual purposed to act as an acclimation status trial and Day 1 of cycle training. Training sessions were conducted during each weekday over a consecutive 3-week span (Days 2–21). This resulted in a total of 14 weekday (no training on weekends) training sessions of 60 min of cycling at a self-selected RPE [26] of 15 (hard), as used previously [11,27], allowing for an applied training approach, as shown in Table 1. Trials were conducted in a temperature- and humidity-controlled environmental chamber. To limit the amount of heat acclimation before the study, training in the heated (33 °C) trials took place during the colder months of the year (January and February) and the room temperature (20 °C) trials took place during more temperate months (April, May, and September).

### 2.2. Fitness Assessments

Fitness assessments were conducted 3 days prior to and 3 days after the 3-week training intervention within room temperature (20 °C) conditions. The use of temperate conditions for pre- and post-testing allowed for direct fitness comparisons between groups, without the acute impact of temperature. Fitness assessments consisted of height (stadiometer, Seca, Chino, CA, USA), weight (Befour PS-660 ST, Saukville, WI, USA), body composition (body fat %), and maximal aerobic capacity (VO_2peak_). To determine body composition, body density was measured via hydrodensitometry in a custom-designed tank and measured via an electronic load-cell based system (Exertech, Dresbach, MN, USA). Participants were asked to void prior to testing and instructed to completely submerge themselves while seated on the scale. After adjusting for estimated residual lung volume, body density was used to estimate body fat % [28]. Peak aerobic capacity was conducted on an electronically braked cycle (Velotron, RacerMate, Seattle, WA, USA) while expired gases were collected via a metabolic cart (ParvoMedics, TrueOne, Metabolic Systems, Sandy, UT, USA). The protocol consisted of an initial 95 W workload and stage increases of 35 W until volitional fatigue. The power associated with the VO_2peak_ (W_peak_) was calculated by multiplying the relative time spent in the final stage by the 35 W-stage interval and adding it to the workload of the previously completed stage. This absolute intensity (50%W_peak_) was used as the absolute fixed intensity for the pre- and post-temperature tolerance trials.

### 2.3. Temperature Tolerance Trials

Temperature tolerance trial procedures have been previously utilized and published in detail previously [11,27]. Briefly, on the first (Day 1) and last day (Day 22) of training, subjects completed a temperature tolerance trial within their assigned temperature condition (20 °C or 33 °C). These trials consisted of cycling at an absolute intensity (50%W_peak_, 95.5 ± 16.5 W) calculated from the pre-training fitness assessment. These fixed-intensity temperature tolerance trials were aimed at the detection of changes related to acclimation. Subjects were instructed to refrain from exercise for the previous 48 h and arrive in a fasted state. Subjects completed a self-reported 24-h dietary log prior to the Day 1 temperature tolerance trial and were instructed to follow the same diet prior to the Day 22 temperature tolerance trial. Nude body weight and the weight of any water ingested were recorded prior to and directly after the exercise bout on a digital scale (Befour PS-660 ST, Saukville, WI, USA) to calculate sweat rate. Core temperature was monitored continuously during exercise via rectal probe (RET-1, Physitemp Instruments, Clifton, NJ, USA) inserted 12 cm past the anal sphincter. Skin temperature was measured continuously and averaged among 2 sites (chest and back) via skin surface probes (SST-1, Physitemp Instruments, Clifton, NJ, USA). Rectal and skin temperatures were recorded via a 4-channel thermometer SD logger (SDL 200, Extech, Nashua, NJ, USA). Heart rate was monitored continuously via a watch and chest strap (V800, Polar Electronics, Lake Success, NY, USA). Muscle biopsies were collected before and 4 h after the completion of the temperature tolerance trials on Day 1 and Day 22.

### 2.4. Daily Aerobic Exercise Training

Subjects completed a 60-min exercise bout on a cycle ergometer (Phantom 3, CycleOps, Madison, WI, USA) within their randomly assigned intervention temperature (20 °C or 30 °C) on days 2–5, 8–12, and 15–19 (weekends off) at an intensity associated with an RPE of 15 (hard) and were encouraged to increase the intensity progressively to induce a natural overload stimulus, as previously utilized [11]. Thus, all participants completed a combined total of 14 RPE-based training sessions and two fixed-intensity temperature tolerance trials on Day 1 and Day 22 (within 20 °C or 33 °C). Body weight was measured before and after each session. Water was provided ad libitum and the volume consumed was recorded to allow for sweat rate calculations. These workloads were self-selected and recorded via an iPad application (Virtual-Training s.r.o., Klecany Czech Republic). Heart rate was monitored continuously via a chest strap and averaged (Polar Electronics, Lake Success, NY, USA). Rectal probes were replaced with tympanic thermometers (Covidien Genius 2, Mansfield, MA, USA) to ease the burden on participants during the duration of the three weeks of cycle training and averaged for the entire 60-min exercise session.

### 2.5. Muscle Biopsies

Muscle biopsies were obtained before and 4 h post-exercise during the pre- and post-training temperature tolerance trials. Muscle biopsies were extracted from the belly of the *vastus lateralis* from each subject using the percutaneous needle biopsy technique with the aid of suction [29]. Briefly, lidocaine (2–4 mL of 1%) was injected under the skin surface and into the muscle fascia before an incision was made and a muscle sample obtained (~100 mg). The Day 1 biopsies were collected on the same leg (separated by ~2 cm) and Day 22 biopsies were obtained from the other leg. Any excess blood or visible connective tissue was removed from muscle samples and quickly placed into Allprotect Tissue Reagent (Qiagen, Valencia, CA, USA). Samples were stored overnight at 4 °C and then transferred to −30 °C for storage and later batch analyses. The 4 h post-exercise timepoint allows time for mRNA expression and is commonly used in the literature, allowing for appropriate comparisons [11,23,24,30].

### 2.6. Acute and Chronic mRNA Quantification

RNA was extracted from skeletal muscle samples using Trizol (Invitrogen, Carlsbad, CA, USA) methodologies, as previously reported [27]. The RNA yield (215 ± 95 ng·μL^−1^) was quantified using a nano-spectrophotometer (nano-drop ND-149 2000, Thermo Scientific, Wilmington, DE, USA) from each sample of muscle tissue (15.7 ± 3.2 mg). The RNA Integrity was measured using an Agilent RNA 6000 Kit and a 2100 Bioanalyzer (Agilent Technologies, Santa Clara, CA, USA) and indicated high quality intact RNA (RIN, 8.2 ± 1.5). First strand cDNA synthesis was achieved from the extracted RNA using Superscript IV LILO master mix with ezDNAase Enzyme (Invitrogen, Carlsbad, CA, USA). qRT-PCR was run in triplicate on a stratagene mx3005p PCR system (Agilent Technologies, Santa Clara, CA, USA) using a two-step protocol. Each 10 µL reaction volume contained 0.5 µL of primer and probe mix (PrimeTime qPCR assay, Integrated DNA technologies, Coralville, IA, USA), 5 µL of qPCR master Mix (Integrated DNA technologies, Coralville, IA, USA), and 4.5 µL of sample adjusted to contain 0.5625 ng·μL^−1^ of cDNA. All probes and primers were designed and obtained by Integrated DNA Technologies (PrimeTime qPCR assay, Coralville, IA, USA) and presented in the Appendix A. Probes and Primes Supplementary File.

Quantification of gene expression was calculated using the 2^−ΔΔCT^ method [31] relative to the appropriate subject-specific geometric mean of five candidate reference genes: *beta-actin (ACTB)*, *glyceraldehyde-3 phosphate dehydrogenase (GAPDH)*, *cyclophilin (CYC)*, *beta-2-microglobulin (B2M)*, and *ribosomal protein S18 (RPS-18)*. NormFinder software [32] was used to determine the most stable reference genes for each subject. If a reference gene, for a given individual, had a stability value of >0.15, it was removed from analysis and the geometric mean of the remaining reference genes was used (mean intragroup variation, 0.053 ± 0.028). Any subject with less than two stable reference genes was removed from analysis (n = 2), as the assumption of stable reference genes could not be established. To determine the acute response to exercise between conditions, the mRNA response at the 4-hour post-exercise time-point was normalized to the pre-exercise sample on Day 1 and Day 22. To determine the effect of the 3-week intervention, Day 22 pre-exercise mRNA was normalized to the Day 1 pre-exercise mRNA.

### 2.7. Statistical Analyses

Two-way, mixed design (time × temperature group) ANOVAs were used to determine differences between fitness assessment data (body composition, aerobic fitness, and peak power), temperature tolerance trial data (gene expression, heart rate, core temperature, skin temperature, and sweat rate), and daily training data (heart rate, power, post-exercise tympanic temperature, and sweat rate). Gene expression data was log transformed prior to statistical analyses to achieve a normal distribution. If Mauchly’s test for sphericity was violated, the Greenhouse-Geisser correction was applied. If a significant F-ratio was detected, Fisher’s protected least significant difference was used to determine differences, appropriate confidence intervals and effect sizes (η_p_^2^, partial squared eta) are reported. SPSS Statistics for Windows (Version 27.0. IBM Corp., Armonk, NY, USA) was used for all statistical analysis. A probability of type I error less than 5% was deemed significant (*p* < 0.05).

## 3. Results

### 3.1. Fitness Assessments

Anthropometric data were similar between the temperature groups (body mass *p* = 0.797; body fat %, *p* = 0.912; fat mass, *p* = 0.941; and fat free mass, *p* = 0.640) and did not change due to training (body mass, *p* = 0.526; body fat %, *p* = 0.501; fat mass, *p* = 0.452; and fat free mass, *p* = 0.860), and no interaction was observed (body mass *p* = 0.300; body fat %, *p* = 0.653; fat mass, *p* = 0.425; and fat free mass, *p* = 0.956). Aerobic capacity (absolute, *p* = 0.024, η_p_^2^ = 0.230, 95% CI [0.315, 3.388]; relative, *p* = 0.021, η_p_^2^ = 0.240, 95% CI [0.315, 3.388]) and peak power increased with training (*p* < 0.001, η_p_^2^ = 0.531, 95% CI [8.734, 22.383]) but were not different between the temperature groups (absolute, *p* = 0.670; relative, *p* = 0.440; and power, *p* = 0.920), and no interaction was observed (absolute, *p* = 0.207; relative, *p* = 0.249; and power, *p* = 0.796). See Table 2.

### 3.2. Temperature Tolerance Trials

The fixed intensity exercise trial also acted as Day 1 of the training intervention and allowed for pre- and post-testing (on Day 22) comparisons at the same absolute intensity within the assigned temperature condition (20 °C or 33 °C). Average heart rates during the 60 min trial were lower for the same power output during the post-intervention trial (*p* < 0.001, η_p_^2^ = 0.691, 95% CI [15.925, 8.353]) but were not different between the temperature groups (*p* = 0.310), and no interaction was observed (*p* = 0.448). Independent of training status, average core (*p* < 0.001, η_p_^2^ = 1.000, 95% CI [1.911, 0.735) and skin (*p* = 0.016, η_p_^2^ = 0.258, 95% CI [0.345, 2.957]) temperatures were higher in the 33 °C group than the 20 °C group. Independent of temperature, Day 22 average skin temperatures decreased from Day 1 (*p* = 0.006, η_p_^2^ = 0.319, 95% CI [1.408, 0.266), but training did not influence core temperature (*p* = 0.598). Interestingly, both core temperature (+0.13 °C in 20 °C group vs. −0.24 °C in 33 °C group; *p* = 0.099, η_p_^2^ = 0.130) and skin temperature (−1.32 °C in 20 °C group vs. −0.35 °C in 33 °C group; *p* = 0.090, η_p_^2^ = 0.137) trended toward interaction but did not reach statistical significance. The 33 °C group had a higher sweat rate than the 20 °C group (*p* = 0.008, η_p_^2^ = 0.303, 95% CI [67.9, 394.9]), but sweat rate was not influenced by training (*p* = 0.571), and no interaction was observed (*p* = 0.224). See Table 3.

### 3.3. Daily Aerobic Exercise Training

**Figure 1:**
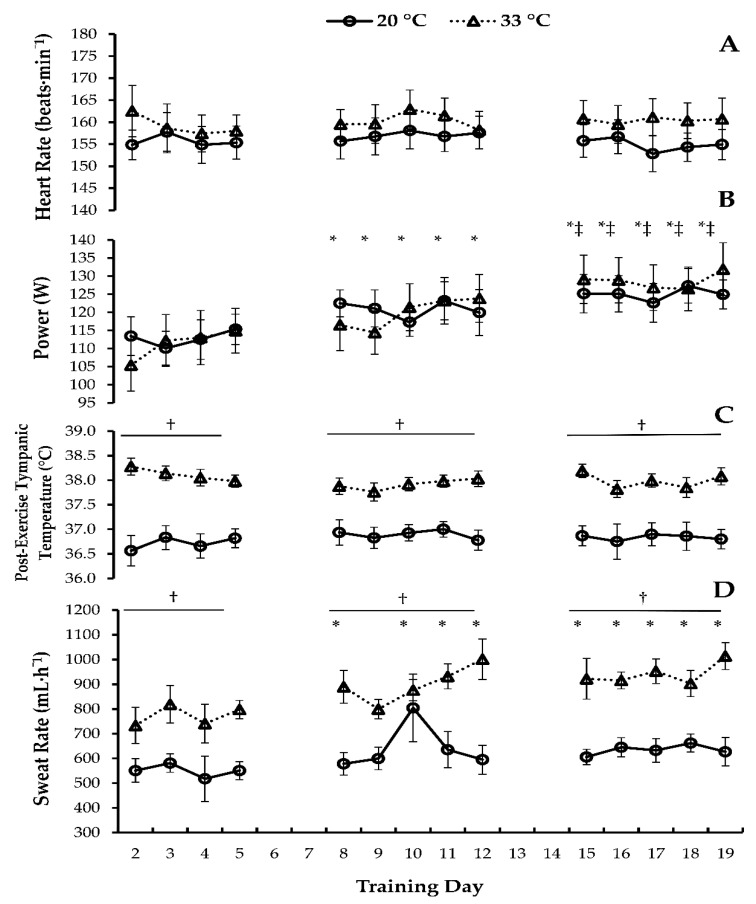
Daily exercise training data. (**A**) heart rate, (**B**) power, (**C**) post-exercise tympanic temperature, and (**D**) sweat rate. 60 minutes of cycle training for a 3-week period, resting on the weekends, within the 20 °C and 33 °C Groups. Data are mean ± SE. * *p* < 0.05 different from Day 2. † *p* < 0.05 different from 20 °C. ‡ *p* < 0.05 different from Day 8. Day 1 (Pre-Trained).

Heart rate did not change over the duration of the progressive-intensity training (*p* = 0.724) and was not different between the temperature groups (*p* = 0.427), and no interaction was observed (*p* = 0.659). The daily self-selected power produced during the cycling sessions increased overtime (*p* < 0.001, η_p_^2^ = 0.347). More specifically, power produced during the daily workouts within weeks 2 and 3 (Days 8–19) were each individually greater than Day 2 (multiple *p* values < 0.004, with a range of 95% CI [27.290, 0.862]), and the daily power outputs for each day within week 3 (Days 15–19) were greater than Day 8 (multiple *p* values < 0.045, with a range of 95% CI [14.797, 0.132]). More importantly, the power output between the temperature groups were not different (*p* = 0.941), and no interaction was observed (*p* = 0.225). Post-exercise tympanic temperature did not change due to training (*p* = 0.798) but was greater in 33 °C than 20 °C (*p* < 0.001, η_p_^2^ = 1.000, 95% CI [0.0679, 1.670]), and no interaction was observed (*p* = 0.244). Sweat rate was calculated after each exercise session. Sweat rates were higher in 33 °C than in 20 °C (*p* < 0.001, η_p_^2^ = 0.544, 95% CI [0.152, 0.378]). Besides Day 9 (*p* = 0.128), all of weeks 2 and 3 (Days 8–19) had higher sweat rates than Day 2 (multiple *p* values < 0.038); however, no interaction was observed (*p* = 0.227). See Figure 1.

### 3.4. Acute and Chronic Exercise mRNA Responses to 33 °C and 20 °C

*PGC-1α* and *VEGF* mRNA increased in response to the acute exercise on Day 1 (*PGC-1α*, *p* < 0.001, 95% CI [1.584, 2.575]; *VEGF*, *p* < 0.001, 95% CI [1.162, 1.936]) and Day 22 (*PGC-1α*, *p* < 0.001, 95% CI [1.068, 1.538], *VEGF*, *p* < 0.001, 95% CI [0.357, 0.970]), and there was no interaction (*PGC-1α*, *p* = 0.405; *VEGF*, *p* = 0.314). However, the 3-week intervention blunted Day 22 mRNA responses, when compared to Day 1 (*PGC-1α*, *p* = 0.004, 95% CI [0.285, 1.268]; *VEGF*, *p* < 0.001, 95% CI [0.461, 1.317]). Temperature did not influence *PGC-1α* (*p* = 0.455) and *VEGF* (*p* = 0.962) mRNA. *NRF1* mRNA decreased in response to the acute exercise on Day 1 (*p* = 0.022, 95% CI [0.060, 0.692]) and Day 22 (*p* = 0.018, 95% CI [0.064, 0.603]) but was not different between days (*p* = 0.842) or temperatures (*p* = 0.517), and there was no interaction (*NRF1*, *p* = 0.621). The acute *GABPA* mRNA response did not change on Day 1 (*p* = 0.234) but decreased in response to acute exercise on Day 22 (*p* = 0.004, 95% CI [0.130, 0.575]). However, the acute *GABPA* mRNA responses were not statistically different between days (*p* = 0.466), nor between temperatures (*p* = 0.688), and there was no interaction (*GABPA*, *p* = 0.175). *TFAM* and *ESRRa* did not change due to acute exercise, between days (*TFAM*, *p* = 0.138; *ESRRa*, *p* = 0.209), or between temperatures (*TFAM*, *p* = 0.788; *ESRRa*, *p* = 0.730), nor were there any interaction effects (*TFAM*, *p* = 0.955; *ESRRa*, *p* = 0.741). See Table 4.

*BNIP3-L* mRNA did not change due to the acute exercise on Day 1 (*p* = 0.093) and Day 22 (*p* = 0.346); however, after the 3-week intervention, Day 22 *BNIP3-L* mRNA was higher than Day 1 (*p* = 0.023, 95% CI [0.102, 1.202]). Temperature did not influence *BNIP3-L* mRNA (*p* = 0.486). *PINK1*, *PARK2*, and *BNIP3* did not change due to the acute exercise, between days (*PINK1*, *p* = 0.571; *PARK2*, *p* = 0.384; *BNIP3*, *p* = 0.571), nor between temperatures (*PINK1*, *p* = 0.855; *PARK2*, *p* = 0.503; *BNIP3*, *p* = 0.906), and there was no interaction (*PINK1*, *p* = 0.268; *PARK2*, *p* = 0.905; *BNIP3*, *p* = 0.930; *BNIP3-L*, *p* = 0.785). See Table 4.

Gene expression for metabolic-related genes were not influenced by temperature (*CS*, *p* = 0.626; *COX4*, *p* = 0.558; *BHAD*, *p* = 0.925), and there was no interaction (*CS*, *p* = 0.751; *COX4*, *p* = 0.447; *BHAD*, *p* = 0.714). *CS*, *COX4*, and *BHAD* mRNA were not influenced by acute exercise on Day 1 (*p* = 0.188, *p* = 0.909, *p* = 0.105). However, after the 3-week training intervention, *COX4* (Day 22; *p* = 0.005, 95% CI [0.108, 0.523]) and *BHAD* (Day 22, *p* = 0.011, 95% CI [0.098, 0.667]) had a blunted response compared to Day 1. There were no differences for *CS* between days (*p* 0.188). See Table 4.

### 3.5. Resting mRNA Responses to Training in 33 °C and 20 °C

The influence of this training and temperature intervention on resting mRNA was determined by comparing the resting biopsy on Day 22 relative to resting on Day 1. Resting *NRF1* and *GABPA* mRNA were lower after training within 20 °C (interaction, *p* = 0.032, 95% CI [0.222, 0.321]; *p* = 0.003, 95% CI [0.340, 1.385), yet no change was detected within the 33 °C (*p* = 0.127, 0.121). Regardless of temperature, resting *PGC-1α* mRNA demonstrated an increased trend overtime (main effect, *p* = 0.057, 95% CI [0.011, 0.615]) on Day 22. Temperature had no influence on resting *PGC-1α* mRNA (*p* = 0.087). Regardless of temperature, resting *VEGF* mRNA was higher on Day 22 (*p* = 0.035, 95% CI [0.041, 0.996]) when compared to Day 1. However, temperature had no influence on resting *VEGF* mRNA (*p* = 0.099). There were no detectable changes to resting mitochondrial biogenesis-related gene expression due to training or temperature for *TFAM* (*p* = 0.162, *p* = 0.125) and *ESRRa* (*p* = 0.931, *p* = 0.228). See Table 5.

For the most part, resting mRNA for mitophagy-related genes *PINK1* (*p* = 0.192, *p* = 0.323), *PARK2* (*p* = 0.969, *p* = 0.651), and *BNIP3* (*p* = 0.137, *p* = 0.173) were not altered due to training or temperature, except for a main effect trend due to training for *BNIP3-L*, suggesting a potential reduction on Day 22 (*p* = 0.087, 95% CI [0.070, 0.953). However, temperature had no influence on resting *BNIP3-L* mRNA (*p* = 0.854). See Table 5.

Regardless of temperature, *CS* and *COX4* resting mRNA were increased on Day 22 (*CS*, *p* = 0.042, 95% CI [0.014, 0.693]; *COX4*, *p* < 0.001, 95% CI [0.432, 1.171) relative to Day 1, but temperature had no effect (*CS*, *p* = 0.231; *COX4*, *p* = 0.124). *BHAD* mRNA did not change with training (*p* = 0.180); nor did temperature (*p* = 0.282). See Table 5.

## 4. Discussion

Historically, “classical” heat acclimation markers (decreased core temperature and increased sweat rate) are based on the physiological changes of males [3,4,5,6,7,8,9,10,11,12,13,14,19], while fewer have investigated heat acclimation in females [7,14]. Therefore, this applied approach to heat acclimation set out to determine the impact of 3 weeks of aerobic exercise in the heat (33 °C) vs. room temperature (20 °C) on thermoregulation, aerobic capacity, and gene expression related to mitochondrial biogenesis, mitophagy, and metabolic development in untrained females. To our knowledge, these are the first methods to examine the influence of heat acclimation on gene expression via muscle tissue in females. Here, we report three main findings: (i) temperature did not influence the Day 1 post-tolerance trial gene expression; (ii) Day 22 post-tolerance trial gene expression responses were attenuated due to training, but only *BNIPL-3* was influence by temperature; and (iii) this 3-week applied heat acclimation exercise routine improved aerobic fitness, peak power, and altered thermoregulation (via reduced skin temperatures). Overall, these results did not detect any temperature group acclimation differences and limited gene expression differences due to the fixed, absolute intensity tolerance trial. As such, an improved training status in both temperature conditions suggests that females may acclimate in a non-classical manner, i.e., differently from males.

Prior to any heat acclimation training, the Day 1 fixed-intensity (50% W_peak_) temperature (33 °C or 20 °C at 40% RH) tolerance trial induced acute increases (*PGC-1α* and *VEGF*) and decreases (*NRF1*) in mitochondrial biogenesis-related mRNA gene expression that were similar between temperature-groups. This suggests the skeletal muscle transcriptional responses were not impeded by the heat in these females. Although the intensity of this fixed-intensity exercise session was moderate, it was sufficient to initiate an upregulated mRNA for *PGC-1α* and *VEGF* within this group of untrained females. Increased *PGC-1α* and *VEGF* mRNA suggest an upregulated stimulus for mitochondrial and angiogenic growth potentials and have been consistently demonstrated after aerobic exercise within our lab and others [11,22,23,24,27,33,34]. More importantly, in these females, ambient heat had no influence over acute mRNA expression beyond that of room temperature. This is contrary to previous works using similar mRNA processing methods [11,22,23,27] that demonstrate consistently blunted mitochondrial-related mRNA (*PGC-1α* and *VEGF*) when performed within heated conditions. One likely explanation for these differences may be the underutilization of female participants within these heat-associated acute exercise projects [11,22,24]. Consequently, these previous post-exercise skeletal muscle gene expression data in men demonstrated an acute heat-associated blunted rise in *PGC-1α* [11,24] and *VEGF* [22] when compared to temperate conditions. Using similar heat acclimation and gene expression methods, this was not the case for these females. Acute *PGC-1α* and *VEGF* mRNA increased similarly among the temperature groups on Day 1. Further, the acute decreases of *NRF1* mRNA were similar among the female temperature groups. This was also different from previous gene expression in males [22], demonstrating an acute *NRF1* downregulation in the heat compared to the room temperature. Together this suggests, prior to any heat acclimation training intervention, these females were more physiologically prepared to endure the heat and their acute gene expression data supports such a claim. Further, the Day 1 temperature tolerance trial was effective at separating the temperature groups via core temperature, skin temperature, and whole-body sweat rates. Therefore, females may physiologically compensate for ambient heat differently from males, independent of acclimation status.

This investigation’s Day 22 attenuated gene expression stimulus echoed fitness assessment improvements (increased VO_2peak_ and W_peak_). An attenuated adaptive stimulus for mRNA expression suggests a reduced need to produce more protein due to training adaptations [33]. A Day 22 post-exercise diminished mRNA expression (*PGC-1α*, *VEGF*, *COX4*, and *BHAD*) represents an abundance of proteins associated with these genes, and thus an attenuated transcriptional stimulus is needed to maintain homeostasis. In applied terms, the absolute intensity on Day 22 no longer provided an overload stimulus. The fixed, absolute intensity of the Day 22 tolerance trial may have contributed to a reduced adaptive stimulus because it did not account for improvements in aerobic fitness but did allow for direct comparison. Resting mRNA gene expression comparisons (Day 22 vs. Day 1), designed to detect alterations due to training, point toward increased mRNA baseline values, as evidence by increased resting mRNA for *VEGF*, *CS*, and *COX4* on Day 22. Previous post-exercise skeletal muscle gene expression data, using similar Day 22 methods, demonstrated a *PGC-1α* acclimation effect in men [11]. In other words, after the 3 weeks of heat acclimation and aerobic training, post-exercise *PGC-1α* mRNA responses were similar between temperate (20 °C) and heated (33 °C) men. Interestingly, non-statistical comparisons indicate that this applied heat acclimation protocol induced similar Day 22 *PGC-1α* relative reductions in mRNA expression, with both sexes decreasing ~2 fold change within 20 °C and ~4 fold change within 33 °C [11]. Therefore, females appear to respond to the heat in a different manner from males, yet the post-trained molecular responses were relatively similar.

Additional Day 22 resting mRNA revealed a reduced *NRF1* and *NRF2* (*GABPA*) expression within 20 °C but not 33 °C, which were the only temperature dependent differences. The proteins encoded from these genes are instrumental cofactors coordinating the response between the nuclear and mitochondrial genome [35]. This suggests that training in temperate conditions may have an added benefit over heated conditions. The applied importance of reduced *NRF1* and *NRF2* (*GABPA*) mRNA of this small magnitude is unclear. Finally, mRNA gene expression for the mitophagy-associated gene *BNIP3-L* increased after the Day 22 tolerance trial. Intuitively, this suggests an improved sensitivity for mitophagy-associated signaling; however, no other mitophagy-related mRNA alterations were detected, including its predecessor of a similar likeness, *BNIP3*. However, attaching too much importance to these limited fold changes in resting mRNA comparisons, without applied functional alterations, should be cautioned against. Especially, considering there were no other mitochondrial-related resting mRNA changes (*PGC-1α*, *TFAM*, *ESRRa*, *PINK1*, *PARK2*, *BNIP3*, and *BNIP3-L*), these data should not be overinterpreted.

As previously demonstrated [11], an RPE-based exercise training approach allowed for consistent increases in power and minimized the potential daily variation in heart rate during the 14 weekday training sessions. This training approach is further validated by the results of these females, who improved their peak power and aerobic fitness independent of the temperature condition. Furthermore, daily post-exercise tympanic temperatures and sweat rates displayed clear separations between the temperature groups. As evidenced by rises in core temperature and sweat rates during training and reduced skin temperatures and on Day 22 (tolerance trial), the 20 °C group most likely experienced some heat acclimation effects. Based on the current data, it appears that this group of females were not hindered by training within the heat (33 °C). Finally, the temperature tolerance trial allowed for pre- and post-testing at the same absolute intensity within the assigned temperature condition (20 °C or 33 °C). Thus, by clamping intensity, any respective alterations due to heat acclimation would be detected. However, unlike the heat acclimation results in men, who displayed increased sweat rates and thus a reduced core temperature [11], these females did not increase their sweat rates during the Day 22 tolerance trial and yet were still able to keep their core temperature in check. The specific mechanisms allowing for such thermoregulation, or “non-classical” acclimation, is not known, but is worthy of further investigation. Previous research suggests that females may require an extended time-period for heat acclimation compared to males [15,21]. Females may even be at a disadvantage within the heat because of increased insulation via a larger percent of body fat and reduced sudomotor function [15,19,20], suggesting greater heat loss requirements. Others point to a smaller body mass and larger body surface to mass ratio (generally found in females) as the cause of a diminished sudomotor response [14,15]. However, even when body attributes were controlled for, heat acclimation differences persisted [17]. Some argue that these heat acclimation differences may, in part, be due to adjustments within female thermoregulation [16,18]. Females may benefit from a wider resting core temperature setpoint and thus may tolerate heat more efficiently than males. Although menstrual cycle phase was not controlled within this investigation, whether this has any influence upon acute/chronic exercise performance is controversial [36]. Indeed, this design, and the conclusions of previous research, make it difficult to separate the effects of exercise training and heat acclimation; however, when compared to previous works of similar methodology in men [11], it is clear that sex differences in the heat do exist, and further investigation is warranted to determine the mechanisms and impact of these differences.

## 5. Conclusions

Here, we report that 3 weeks of exercise and heat acclimation training in untrained females did not hinder applied performance outcomes in comparison to room temperature conditions. Specifically, this applied approach to heat acclimation suggests that (i) temperature did not influence the pre-training acute exercise gene expression, (ii) there was some post-training attenuated gene expression responses attributable to the improved fitness, and (iii) this 3 week-routine improved aerobic fitness, peak power, and altered skin thermoregulation. Therefore, while this investigation did not demonstrate many classical heat acclimation temperature and exercise statistical differences, when placed in the context of previous similar work in males [11,22], these findings become more relevant. Taken together, these conclusions support the notion that sex has an influential role over the physiological acceptance of heat acclamation alterations. Future research is needed to clarify why women seem to be more resilient within the heat and exactly how females manifest these adaptations differently.

From a practical standpoint, caution should be taken when making exercise prescription and work physiology, as well as military training and operations recommendations that are not sex-specific. Although these females were untrained, they were better equipped to deal with the heat than untrained males. Leaders in their respective fields should tailor their heat-associated recommendations to take advantage of this unknown heat-resilient physiological mechanism within females. This can be especially useful where ambient conditions limit the ability for evaporative cooling via sweating (i.e., elevated relative humidity). However, one must be cautioned, these results are based upon controlled settings and do not account for all factors that impact the heat index intensity.

## Figures and Tables

**Table 1:** Experimental Design.

Day	−3	1 *	2–21	22 *	25
Trial	Fitness Assessment	Temperature Tolerance Trial	RPE-based Cycle Training	Temperature Tolerance Trial	Fitness Assessment

Note: Day 1 was dual purposed as the start of cycle training. * Pre- and 4 h Post-exercise biopsies.

**Table 2:** Fitness Assessment Data (20 °C).

	Day –3 (Pre-Trained)			Day 25 (Post-Trained)		
	20 °C Group (n = 12)	33 °C Group (n = 10)	Combined (n = 22)	20 °C Group (n = 12)	33 °C Group (n = 10)	Combined (n = 22)
Body mass (kg)	65.4 ± 11.9	66.8 ± 6.9	66.0 ± 9.8	65.5 ± 11.7	66.4 ± 7.2	65.9 ± 9.7
Body fat (%)	25.5 ± 7.6	26.0 ± 8.1	25.7 ± 7.6	25.4 ± 8.0	25.5 ± 8.1	25.5 ± 7.9
Fat mass (kg)	17.3 ± 8.3	17.8 ± 6.9	17.5 ± 7.5	17.3 ± 8.6	17.4 ± 6.6	17.4 ± 7.6
Fat free mass (kg)	48.0 ± 5.2	48.9 ± 3.3	48.5 ± 4.3	48.1 ± 4.5	49.0 ± 3.8	48.5 ± 4.1
VO_2_peak (L·min^−1^)	2.56 ± 0.35	2.57 ± 0.34	2.57 ± 0.34	2.74 ± 0.34	2.62 ± 0.28	2.69 ± 0.31 *
VO_2_peak (mL·kg^−1^·min^−1^)	40.1 ± 7.0	38.8 ± 6.2	39.5 ± 6.5	42.8 ± 7.0	39.8 ± 4.9	41.5 ± 6.2 *
Peak power (W_peak_)	191 ± 29	190 ± 39	191 ± 33	207 ± 22	205 ± 33	206 ± 27 *

Data are mean ± SD. * *p* < 0.05 from Pre-Trained.

**Table 3:** Fixed-Intensity Temperature Tolerance Trial Data (20 °C or 33 °C).

	Day 1 (Pre-Trained)			Day 22 (Post-Trained)		
	20 °C Group (n = 12)	33 °C Group (n = 10)	Combined (n = 22)	20 °C Group (n = 12)	33 °C Group (n = 10)	Combined (n = 22)
Heart rate (beat·min^−1^)	148 ± 14	156 ± 16	152 ± 16	138 ± 13	142 ± 12	140 ± 13 *
Core temperature ( °C)	37.86 ± 0.92	39.37 ± 0.46 †	38.55 ± 1.06	37.99 ± 0.78	39.13 ± 0.47 †	38.50 ± 0.87
Skin temperature ( °C)	34.44 ± 2.38	35.61 ± 0.89 †	34.97 ± 1.91	33.12 ± 1.52	35.26 ± 0.88 †	34.09 ± 1.65 *
Sweat rate (mL·h^−1^)	469 ± 185	692 ± 296 †	571 ± 261	423 ± 269	662 ± 192 †	532 ± 262

Data collected from Day 1 and Day 22 Temperature Tolerance Trials. Core temperature was recorded via rectal probe. Data are mean ± SD. * *p* < 0.05 from Pre-Trained. † *p* < 0.05 from 20 °C.

**Table 4:** Acute and Chronic Relative Gene Expression on Day 1 and Day 22.

	Day 1 (Pre-Trained)			Day 22 (Post-Trained)		
	20 °C Group (n = 12)	33 °C Group (n = 10)	Combined (n = 22)	20 °C Group (n = 12)	33 °C Group (n = 10)	Combined (n = 22)
**Mito. Biogenesis**						
* PGC-1α*	4.77 ± 2.44	5.92 ± 0.70	5.29 ± 3.29 *	3.07 ± 1.09	2.23 ± 0.77	2.69 ± 1.03 *†
* NRF1*	0.86 ± 0.49	0.87 ± 0.41	0.86 ± 0.45 *	0.94 ± 0.37	0.79 ± 0.36	0.87 ± 0.37 *
* GABPA* (*NRF2*)	0.94 ± 0.51	0.99 ± 0.29	0.96 ± 0.42	0.94 ± 0.29	0.73 ± 0.29	0.84 ± 0.31 *
* VEGF*	3.02 ± 1.54	3.93 ± 2.85	3.44 ± 2.22 *	1.94 ± 0.97	1.65 ± 1.23	1.81 ± 0.23 *†
* TFAM*	1.10 ± 0.49	1.16 ± 0.61	1.13 ± 0.53	0.96 ± 0.44	0.81 ± 0.01	0.89 ± 0.33
* ESRRa*	0.86 ± 0.32	1.29 ± 1.08	1.06 ± 0.77	0.92 ± 0.35	0.87 ± 0.35	0.89 ± 0.35
**Mitophagy**						
* PINK1*	0.87 ± 0.72	1.57 ± 1.60	1.19 ± 1.23	1.20 ± 0.71	0.86 ± 0.44	1.05 ± 0.62
* PARK2*	1.22 ± 1.17	2.12 ± 2.34	1.63 ± 1.81	0.85 ± 0.50	1.06 ± 0.66	0.94 ± 0.57
* BNIP3*	1.05 ± 0.60	1.37 ± 1.18	1.19 ± 0.90	1.06 ± 0.73	0.85 ± 0.30	0.97 ± 0.57
* BNIP3-L*	0.96 ± 0.59	0.94 ± 0.61	0.95 ± 0.59	1.56 ± 1.02	1.30 ± 1.81	1.44 ± 1.08 †
**Metabolic**						
* CS*	0.92 ± 0.55	1.23 ± 1.06	1.06 ± 0.81	0.90 ± 0.39	0.84 ± 0.26	0.87 ± 0.33
* COX4I1* *(COX4)*	1.01 ± 0.28	1.22 ± 0.86	1.11 ± 0.61	0.93 ± 0.31	0.75 ± 0.18	0.85 ± 0.27 *
* HADH* *(BHAD)*	0.82 ± 0.35	1.08 ± 0.81	0.94 ± 0.60	0.95 ± 0.61	0.75 ± 0.24	0.86 ± 0.48 *

Data are mean ± SE. Relative fold change in genes of interest normalized to Pre-Exercise (fold change of 1.0) and reference genes *ACTB*, *GAPDH*, *CYC*, *B2M*, and *RPS18*. * *p* < 0.05 different from Pre-Exercise (fold change of 1.0). † *p* < 0.05 different from Day 1.

**Table 5:** Resting mRNA on Day 22 Relative to Day 1.

	20 °C Group (n = 12)	33 °C Group (n = 10)	Combined (n = 22)
**Mito. Biogenesis**			
* PGC-1α*	1.12 ± 0.19	1.71 ± 0.28	1.39 ± 0.17
* NRF1*	0.80 ± 0.08 *	1.37 ± 0.21	1.06 ± 0.12
* GABPA* (*NRF2*)	0.87 ± 0.09 *	1.67 ± 0.26	1.24 ± 0.15
* VEGF*	1.14 ± 0.11	3.95 ± 2.37	2.42 ± 1.09 *
* TFAM*	1.10 ± 0.20	1.53 ± 0.28	1.30 ± 0.17
* ESRRa*	0.89 ± 0.07	1.52 ± 0.41	1.18 ± 0.19
**Mitophagy**			
* PINK1*	1.45 ± 0.37	3.52 ± 2.18	2.39 ± 1.01
* PARK2*	1.10 ± 0.24	2.72 ± 1.86	1.84 ± 0.85
* BNIP3*	1.08 ± 0.10	2.22 ± 0.79	1.60 ± 0.38
* BNIP3-L*	0.81 ± 0.14	1.13 ± 0.30	0.95 ± 0.16
**Metabolic**			
* CS*	1.17 ± 0.12	1.91 ± 0.60	1.51 ± 0.28 *
* COX4I1* *(COX4)*	1.51 ± 0.16	3.02 ± 1.17	2.20 ± 0.55 *
* HADH* *(BHAD)*	1.13 ± 0.12	1.73 ± 0.52	1.40 ± 0.24

Data are mean ± SE. Data are expressed as Pre-exercise gene expression on Day 22 relative to Pre-exercise on Day 1 (fold change of 1.0). * *p* < 0.05 different from Day 1.

## Data Availability

Please request the datasets from this investigation from the corresponding author.

## Appendix A

**Table A1:** Probes and Primers Supplementary Data.

Reference Genes	Primer 1	Primer 2	Probe
* ACTB*	AAGTCAGTGTACAGGTAAGCC	GTCCCCCAACTGAGATGTATGT	CTGCCTCCACCCACTCCCA
* B2M*	ACCTCCATGATGCTGCTTAC	GGACTGGTCTTTCTATCTCTTGT	CCTGCCGTGTGAACCATGTGACT
* CYC*	TCTTTCACTTTGCCAAACACC	CATCCTAAAGCATACGGGTCC	TGCTTGCCATCCAACCACTCAGTC
* GAPDH*	TGTAGTTGAGGTCAATGAAGGG	ACATCGCTCAGACACCATG	AAGGTCGGAGTCAACGGATTTGGTC
* RSP18*	GTCAATGTCTGCTTTCCTCAAC	GTTCCAGCATATTTTGCGAGT	TCTTCGGCCCACACCCTTAATGG
**Mitochondrial Biogenesis**			
* PGC-1α*	GCAATCCGTCTTCATCCACA	CCAATCAGTACAACAATGAGCCT	AGCAGTCCTCACAGAGACACTAGACAG
* NRF1*	GTCATCTCACCTCCCTGTAAC	GATGCTTCAGAATTGCCAACC	ATGGAGAGGTGGAACAAAATTGGGC
* GABPA (NRF2)*	TGTAGTCTTGGTTCTAGCAGTTTC	TGGAACAGAGAAAGCAGAGTG	TGGTTCATTGATGTCTATGGCCTGGC
* VEGF*	GCGCTGATAGACATCCATGA	CCATGAACTTTCTGCTGTCTTG	TGCTCTACCTCCACCATGCCAAG
* TFAM*	GCCAAGACAGATGAAAACCAC	TGGGAAGGTCTGGAGCA	CGCTCCCCCTTCAGTTTTGTGTATTT
* ESRRa*	TCTCCGCTTGGTGATCTCA	CTATGGTGTGCATCCTGTG	TGGTCCTCTTGAAGAAGGCTTTGCA
**Mitophagy**			
* PINK1*	GTTGCTTGGGACCTCTCTTG	TGAACACAATGAGCCAGGAG	TGTAAGTGACTGCTCCATACTCCCCA
* PARK2*	GCTTGGTGGTTTTCTTGATGG	TTGAAGCCTCAGGAACAACT	CCTGCTCGGCGGCTCTTTCA
* BNIP3*	CCACTAACGAACCAAGTCAGAC	CATCTCTGCTGCTCTCTCAT	AAAGGTGCTGGTGGAGGTTGTCA
* BNIP3-L*	CAAACATGATCTGCCCATCTTC	TCCTCATCCTCCATCCACAA	TCTCACTGTGACAGCCCTTCGC
**Metabolic Enzymes**			
* CS*	GTAGCAGTTTCTGGCATTCAG	ACTGTGGACATGATGTATGGTG	TGAGAGGCATGAAGGGATTGGTCTATGA
* COX4I1 (COX4)*	AGTCTTCGCTCTTCACAACA	GCAGTGGCGGCAGAATG	AGGTGGAAATTGCTCGCTTGCC
* HADH (BHAD)*	CAAGTTTCATGACAGGCACTG	AAGGCTGGACAAGTTTGCT	CCACCAGACAAGACCGATTCGCT

ACTB, β-actin; B2M, 2-microglobulin; CYC, cyclophilin; GAPDH, glyceraldehyde-3-phosphate dehydrogenase; RPS18, ribosomal protein S18; PGC-1α, peroxisome proliferator-activated receptor-γ coactivator 1α; NRF1, nuclear respiratory factor 1; GABPA, GA-binding protein-α; VEGF, vascular endothelial growth factor; TFAM, mitochondrial transcription factor A; ESRRα, estrogen-related receptor-α; PINK1, PTEN-induced kinase-1; PARK2, parkin an E3 ubiquitin ligase; BNIP3, Bcl2-interacting protein-3, BNIP3-L, Bcl2-interacting protein 3-like; CS, citrate synthase; COX4I1, cytochrome C oxidase subunit 4I1; HADH, hydroxyacyl-coA dehydrogenase.
